# Stability in attention-deficit hyperactivity disorder prevalence: wishful illusion or complex reality?

**DOI:** 10.1192/bjo.2026.11009

**Published:** 2026-03-30

**Authors:** Yasser Saeed Khan, Waleed Ahmed, Jigna Stott

**Affiliations:** Mental Health Service, Hamad Medical Corporation, Doha, Qatar; College of Medicine, Qatar University, Doha, Qatar; Maudsley Health, Abu Dhabi, UAE; Psychiatry UK, Camelford, UK

**Keywords:** Attention-deficit hyperactivity disorder, neuropsychiatry, diagnosis and classification, epidemiology, neurodevelopmental disorders

## Abstract

This editorial examines the current debate surrounding attention-deficit hyperactivity disorder prevalence, the perceived surge in diagnoses and the growing pressure on healthcare services. It discusses the wide methodological variation in recent studies, the limited pool of high-quality evidence and the challenges this creates when trying to understand true population rates. The article highlights the gap between stable epidemiological estimates and the marked rise in referrals, waiting lists, private assessments and prescribing. It explores how increased awareness, evolving diagnostic criteria and improved detection of previously unrecognised cases contribute to the overall picture, along with the role of social media and shifting societal attitudes. Implications for policy and clinical practice are outlined, emphasising the need for efficient clinical pathways, better-quality data and more comprehensive, multi-informant assessments.

**Figure:**
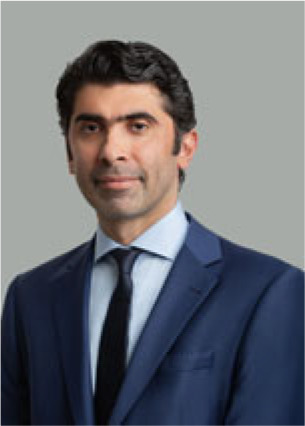

A recently published systematic review, ‘The changing prevalence of ADHD? A systematic review’, led by Martin and colleagues at King’s College London,^1^ aims to clarify the post-2020 landscape of attention-deficit hyperactivity disorder (ADHD) prevalence. It systematically analysed 40 studies across 17 countries. The purpose of the review was to update global estimates and assess the quality of the evidence base. The authors deserve commendation for carrying out a timely and pertinent review, given the global public and clinical uproar regarding a perceived surge in diagnoses.^2,3^ The authors’ rigorous adherence to the Preferred Reporting Items for Systematic reviews and Meta-Analyses guidelines and their transparent reporting of methodological challenges are worthy of appreciation. However, while appreciating methodological integrity, we wish to offer a critical perspective on the review’s interpretation of findings and its broader implications.

## Navigating the prevalence labyrinth

The central conclusion of the review, that the highest-quality findings do not suggest an increase in prevalence since 2020 but indicate some variability in incidence during the COVID-19 pandemic,^1^ warrants further scrutiny. The review acknowledges significant heterogeneity across studies, particularly those involving paediatric patients, deeming a pooled meta-analysis inappropriate. This extreme variation implies that the underlying studies are fundamentally different in their methodologies and populations. Therefore, indicating a conclusion about ‘prevalence’ may be potentially misleading.^4^

A critical limitation, candidly highlighted by the authors, was that the generally poor quality of the included literature, with over 70% of studies included having a high or very high risk of bias. This leaves only four studies deemed ‘low risk of bias’ to support the primary conclusion regarding stable prevalence. Among these studies, one retrospective cohort study in Ontario, Canada analysed the electronic medical records of over 288 000 children and youth.^5^ Two studies documented parent-reported ADHD diagnoses from nationally representative US surveys that included 26 000 and 37 000 children, respectively.^6,7^ The only low-risk study to include adults, in addition to children, utilised data from the Swedish National Patient Registers, providing insights across the lifespan.^8^

Although focusing on high-quality evidence is a strength, the extremely limited number of such studies clearly restricts the generalisability and robustness of any inferences about global prevalence trends. To base a conclusion of no significant rise predominantly on four studies, however robust, within a field characterised by such vast methodological inconsistencies, may present an overly conservative and potentially incomplete picture. The review notes that estimates in these studies ranged markedly, even from biased studies, reinforcing the challenge of drawing any firm conclusions.

Furthermore, the review details the different modalities of reporting ADHD symptoms, which included self-report, electronic medical records and, less frequently, diagnostic interviews. It correctly points out that rates were consistently higher when ADHD was assessed by self-report surveys compared with diagnostic interviews, and that diagnostic interview rates were in turn higher than existing diagnosis rates (i.e. recorded diagnoses in a clinician-diagnosed population). This inherent variability based on assessment method is a crucial factor. If self-report surveys, which the authors deem to carry a higher risk of bias, consistently yield higher estimates, then a conclusion of stable ‘prevalence’ based on a limited number of medical record studies might reflect the stability of only diagnosed cases within specific healthcare systems, rather than the true underlying population prevalence, or indeed the growing awareness and identification of ADHD symptoms. It is important to note that this pattern is not unique to ADHD, because similar discrepancies have been observed in other conditions including depression, where self-report surveys often yield higher prevalence estimates than clinician-administered interviews.^9^

## The disconnect between perceived surge and actual prevalence

Perhaps the most salient points for critical reflection lie in the limitations section of the published review, which reiterates its own interpretative challenges. The authors explicitly state a profound gap – the absence of data reporting ADHD referrals, waiting lists or use of private assessments, as well as data on ADHD within educational settings – despite reports of systems being overwhelmed.^10–12^ NHS England shows a 13% year-on-year rise in referrals, now reaching around 20 000 new referrals each month.^13^ Furthermore, national prescribing figures show a remarkable 51% increase in the number of patients receiving ADHD medication between 2019/20 and 2022/23.^10^

The initial motivation for the review seemed to have originated from clinicians, teachers and charities reporting increasing demand for ADHD assessments in recent years. The absence of empirical data on these reported increases in demand (referrals, waiting lists and private assessments) means that the review cannot truly address the observed ‘surge’ in public and clinical perception, even if that surge does not translate to an increase in ‘true’ underlying prevalence. The conclusion that this does not necessarily indicate an increase in ADHD prevalence is accurate in a strict epidemiological sense of underlying rates, but the very real and pertinent issue of escalating demand on services cannot be overlooked.

The authors rightly highlight the significant delay between data collection and publication, with many screened studies being excluded for containing pre-2020 data despite recent publication. This fundamental flaw in research dissemination means that even the most current systematic reviews often include data that are already several years old. This further complicates the assessment of rapid changes in prevalence or incidence.

Although the review states that its findings surrounding true prevalence cannot explain any increase in diagnostic referrals, it later offers a compelling alternative. The increase in ADHD assessments may indeed reflect a societal change in how the condition is conceptualised, with greater awareness about neurodiversity.^14^ This shift, coupled with changes in diagnostic criteria (e.g. DSM-5 lowering symptom requirements for adults, increasing age-of-onset threshold),^15,16^ is a vital component of the prevailing ADHD narrative. Although the review’s focus on ‘prevalence’ as a static epidemiological measure is important, it might underemphasise the dynamic interplay of increased awareness, evolving diagnostic frameworks and improved detection of previously undiagnosed individuals. The limited geographical and cultural representativeness of high-quality prevalence studies also deserves attention, because much of the evidence base is Western-centric and recent work suggests that rates in the Middle East and North Africa may be higher.^17^

The wide range of prevalence estimates reported by studies conducted in different regions reflects a combination of regional, methodological and sampling differences. For example, survey-based studies from parts of Asia and the Middle East have frequently reported higher prevalence estimates, whereas register-based data from North America and Europe have tended to fall at lower levels. These regional differences, alongside variation in assessment method and study design, suggest how estimates from non-Western regions may diverge due to sample selection, case ascertainment methods and cultural or service-related factors, rather than necessarily reflecting true epidemiological differences.

It is also important to note that the prevalence estimates of ADHD could be influenced by certain co-occurring conditions. For example, autism spectrum disorder, intellectual disability and anxiety disorders can present with overlapping features, making accurate diagnosis of ADHD quite challenging. This diagnostic complexity may affect both population-level estimates and observed clinical demand, stressing further the importance of robust, multi-informant assessments and careful differentiation in both research and clinical practice.

## Implications for clinical practice and policy

The systematic review’s findings carry significant implications for how healthcare systems and policy-makers respond to the escalating demand for ADHD services. The distinction between a rise in true prevalence and an increase in awareness and diagnosis requires effective resource allocation. Rather than planning for an ADHD epidemic, the focus should shift to enhancement of existing pathways to accurately identify and support those who genuinely meet diagnostic criteria. The current long waiting lists and medication shortages are undeniable crises.^18,19^ These issues arise from multiple factors including increased awareness, systemic under-resourcing and, perhaps, an over-reliance on self-reporting, rather than a sole, marked increase in the actual prevalence.

Recent policy developments recognise that rising demand for ADHD services is potentially driven by unmet need and system pressures rather than by a sudden change in underlying prevalence. In England, the 2025 NHS ADHD Taskforce Final Report^20^ sets out recommendations aimed at improving access and support across health, education, employment and justice settings, with an emphasis on pathway redesign, workforce development, improved data quality and greater consistency of care. Professional bodies, including the Royal College of Psychiatrists, have welcomed the report while highlighting ongoing challenges related to workforce capacity, variability in assessment practice and equitable access.

Policy must prioritise the reinforcement of robust, gold-standard diagnostic practices to effectively address the evolving landscape of diagnoses. Healthcare services must emphasise and invest in comprehensive, clinician-led multi-informant, multi-setting assessments and move away from potentially biased single-clinician assessments or reliance on online surveys. Alongside this, strategic resource mobilisation and allocation are crucial. Much of the increased demand appears to arise from previously undiagnosed individuals, including adults, who may have gone ‘below the radar’ for years by ‘masking’ their symptoms. Masking is characterised by the conscious or unconscious use of compensatory strategies to conceal symptoms. These strategies can often lead to burnout, increased anxiety and emotional or physical exhaustion. Masking behaviours have been observed more frequently in females.^21^

This challenge needs to be met through targeted training of primary care professionals and more efficient deployment of specialist resources. Finally, increased diagnoses necessitate holistic and diversified biopsychosocial support. A broader range of evidence-based interventions beyond medication alone must be offered. This should include detailed psychoeducation, behavioural therapies and workplace or educational adaptations, meaning that we ensure improved quality of life, not just provide a label.

## Future directions

The systematic review clearly identifies a critical research gap in the lack of recent, high-quality, population-level epidemiological studies of ADHD prevalence. Future research must prioritise rigorous methodologies, combining clinical assessment with robust sampling strategies for accurate capturing of more reliable prevalence rates. Longitudinal studies are also vital, because these may differentiate between new cases and the ongoing detection of existing, previously undiagnosed cases. International collaboration could further standardise diagnostic practices and research methodology, allowing for more meaningful comparisons of the ADHD global landscape.

In conclusion, Martin and colleagues provide a valuable, methodologically sound systematic review that highlights the urgent need for higher-quality, real-time epidemiological data on ADHD. Although their findings suggest stability in ‘true prevalence’ based on the limited, highest-quality evidence, the review points to a more complex reality. This critical perspective must acknowledge the huge methodological challenges in this field. We advocate for future research and policy discussions to differentiate between ‘true epidemiological prevalence’ and ‘diagnosed prevalence’, given that the latter is influenced by societal awareness, evolving diagnostic criteria and healthcare access. The role of social media is crucial in increasing public awareness of ADHD and encouraging help-seeking behaviours. Nevertheless, the ‘surge’ in demand, waiting lists and public discourse, even if not reflecting a true increase in underlying prevalence, is a major public health phenomenon. It demands further investigation, incorporating data on referrals and service capacity, to truly understand the changing landscape of ADHD detection, demand and support.

## Data Availability

Data availability is not applicable to this article because no new data were created or analysed.
